# Dysbiotic drift: mental health, environmental grey space, and microbiota

**DOI:** 10.1186/s40101-015-0061-7

**Published:** 2015-05-07

**Authors:** Alan C Logan

**Affiliations:** CAMNR, 23679 Calabasas Road Suite 542, Calabasas, CA 91302 USA

## Abstract

Advances in research concerning the mental health implications of dietary patterns and select nutrients have been remarkable. At the same time, there have been rapid increases in the understanding of the ways in which non-pathogenic microbes can potentially influence many aspects of human health, including those in the mental realm. Discussions of nutrition and microbiota are often overlapping. A separate, yet equally connected, avenue of research is that related to natural (for example, green space) and built environments, and in particular, how they are connected to human cognition and behaviors. It is argued here that in Western industrial nations a ‘disparity of microbiota’ might be expected among the socioeconomically disadvantaged, those whom face more profound environmental forces. Many of the environmental forces pushing against the vulnerable are at the neighborhood level. Matching the developing microbiome research with existing environmental justice research suggests that grey space may promote dysbiosis by default. In addition, the influence of Westernized lifestyle patterns, and the marketing forces that drive unhealthy behaviors in deprived communities, might allow dysbiosis to be the norm rather than the exception in those already at high risk of depression, subthreshold (subsyndromal) conditions, and subpar mental health. If microbiota are indeed at the intersection of nutrition, environmental health, and lifestyle medicine (as these avenues pertain to mental health), then perhaps the rapidly evolving gut-brain-microbiota conversation needs to operate through a wider lens. In contrast to the more narrowly defined psychobiotic, the term eco-psychotropic is introduced.

## Introduction

For the last half-century, the World Health Organization and leading psychiatric associations have been underscoring the reality that without mental health, there can be no true physical health [1]; in its condensed form, this message is simply ‘No Health without Mental Health’ [2]. There is little question that mental health disorders represent one of the most important public health issues of our time [3,4]. Major depressive disorder (MDD) and anxiety disorders represent debilitating and potentially life-threatening illnesses that are continuing to rise through the ranks of the global burden of diseases [5-7]. Beyond their direct effects, mental disorders contribute to an epidemic of co-morbidity in the form of other non-communicable diseases such as type II diabetes, cardiovascular disease, obesity, and dementia [8,9].

Over the last decade, there has also been increased awareness by both researchers and clinicians that individuals with subthreshold mental health disorders make up a sizeable portion of patients encountered in primary care settings and that the reported levels of psychological distress, disability in daily activities, and perceived health is often comparable to patients with diagnosable mental disorders [10]. Research shows that those who sit in the subthreshold range of depression are on a trajectory toward higher risk of MDD, dysthymia, social phobia, and generalized anxiety disorder (GAD) [11]. Whether an individual transitions to MDD or not, subthreshold depression is associated with increased demand for healthcare services, non-communicable disease (NCD) co-morbidity, and impaired quality of life [12,13].

It is emphasized here in the introduction that there is much more to mental health than the absence of checklist criteria as found in the Diagnostic and Statistical Manual of Mental Disorders (DSM) and the International Classification of Diseases (ICD). The World Health Organization describes mental health not by the absence of a mental health *disorder* - rather it is ultimately defined as the ability of an individual to reach their potential in the context of physical and social well-being [14]. The ‘No Health without Mental Health’ mantra is therefore not a clever marketing slogan; it should resonate through all aspects of health promotion.

### Roadmap to the current review

The discipline of physiological anthropology strives to understand the ways in which the modern environment exerts selective pressures on humans, and to what extent those pressures influence physiology and ultimately health and well-being. A growing body of research suggests that modern environmental forces, particularly those that may detract from reaching optimal mental health, are not ‘distributed’ equally across populations. In addition to understanding how specific variables might influence physiology (for example, dietary patterns or aspects of the built environment), there is a need to explore collective or synergistic pressures and in whom those collective pressures most likely push upon.

The primary destination of the current review is toward the argument that the environmental forces with which vulnerable populations are confronted - many of them at the neighborhood level - might allow dysbiosis to be the norm rather than the exception in those at risk of suboptimal mental health. Dysbiosis, currently defined, involves perturbations to the structure of complex commensal microbial communities. It is a state of change that involves the loss of beneficial microorganisms, and/or the expansion of potentially harmful microbes, and/or the loss of overall microbial diversity [15].

Generally, dysbiosis is accepted to be a shift away from the microbial composition found in ‘otherwise healthy adults’. However, just because a healthy adult is accepted as a control subject in various studies (for example, inflammatory bowel disease), it does not mean that they possess the ideal microbial ecosystem. Emerging studies concerning the oral microbiota of our ancestors [16], or the fecal microbiota of our modern relatives that etch out a very traditional, low technological existence [17], have forced questions concerning what truly defines dysbiosis. From the perspective of physiological anthropology, dysbiosis in a modern Westernized nation may indeed be a relative term.

The focus throughout the current discussion will be depressive symptoms and psychological distress. In order to highlight how a ‘disparity of microbiota’ might unfold, a process referred to later as dysbiotic drift, more detailed discussions of the pathophysiology of depression, the quality of the residential environment (particularly the urban built environment), dietary patterns, and other socioeconomic determinants will be necessary. Central to the unfolding argument will be the emerging evidence suggesting the intestinal (and overall) microbiome - the microbial communities that occupy habitats on and within the body - can shape some degree of mood and behavior in rodents and humans.

The discussion will include the ways in which specific environmental factors might interact with psychological distress in vulnerable populations. Since microbiota can be shaped by environmental forces, it seems reasonable to ask how socioeconomic disparities might influence the microbiota of those that are already in the highest risk category for depression. Although the review will focus largely on depression, the relevance of the discussion may extend to mental health as the WHO defines the term. The reader is encouraged to view the discussion from the vantage point of prevention and early intervention. The lens of prevention can help magnify the importance of various environmental forces that continually push against high-risk populations.

### Environmental determinants

The etiological heterogeneity of depression has been well-described. Although depression can be experienced by any individual, regardless of socioeconomic circumstance, it is a complicated, multi-factorial illness that does not occur randomly in the population. The risk is far higher among the socioeconomically disadvantaged and generally follows a socioeconomic gradient wherein the highest burden of disease is carried by the most impoverished groups [18-22]. Major negative life events have emerged as a distinct neighborhood-health pathway by which context influences risk of depression [23]. On the other hand, daily hassles are also associated with the provocation of depression [24-26], while neighborhood conditions and a collection of chronic lower-grade negative events also push against socioeconomically disadvantaged populations [27-29]. Research indicates that the link between socioeconomic factors and depression may be particularly strong for persistent, chronic depression [30].

Prenatal and early-life stress and other environmentally mediated adversities initiate epigenetic alterations, which in turn may influence lifetime risk of depressive disorders among offspring. Moreover, these environmental factors may also push trans-generational influences via epigenetic inheritance [31]. Both cumulative disadvantage and current socioeconomic hardship are strongly associated with depressive symptoms in adults [32,33]. Research involving twins shows that education, income, and upward social mobility is associated with lowered risk of depression [34].

Social inequalities are also apparent when researchers break down specific psychological assets that otherwise support positive mental health. Optimism in particular has been consistently linked with socioeconomic advantage [35]. In turn, optimism is associated with healthy lifestyle habits [36] and is an independent predictor of good mental health in urban residents [37]. With so much at stake, there is an urgent and obvious need for a deeper understanding of environmental, genetic, and epigenetic determinants of depressive disorders [38].

### Pathophysiology

Over time, the collective exposure to physical, social, and psychological stressors can manifest in physiological wear and tear known as allostatic load (AL). The provocation of compensatory physiological mechanisms (for example, immune, cardiovascular, neuroendocrine responses that work well in acute situations) on a repetitive basis can lead to multisystem damage. The AL theory helps to explain how chronic stress can lead to physiological dysregulation and subsequent disease [39,40]. In particular, the wear and tear may have detrimental effects on mitochondria [41], the proper functioning of which is intimately connected to mental health [42].

In a bi-directional fashion, AL predicts compromised mental health [43-46], while individual and area-level socioeconomic disadvantage predicts higher levels of AL [47-54]. Lifestyle habits such as regular exercise, tobacco abstinence, and healthy dietary patterns are associated with lower AL [55-57]; however, as described in more detail later, these behaviors may be shaped at the neighborhood level.

Despite the etiological heterogeneity of depression, an increasingly robust body of research indicates that there are clear biological dysregulations associated with depressive symptoms and the diagnosis of MDD. These dysregulations are virtually identical to that associated with AI. They include those involving immuno-inflammatory (for example, elevations in C-reactive protein and inflammatory cytokines), metabolic (for example, insulin resistance, metabolic syndrome), the burden of oxidative stress, hypothalamic-pituitary-adrenal (HPA) axis (for example, cortisol perturbations), neurotransmitter/neuropeptide (for example, dopamine, serotonin, gamma-aminobutyric acid, brain-derived neurotrophic factor) communication, and other systems [58,59]. Low-grade inflammation is a central component of emerging psychiatric research because it can help explain the primary biological disturbances currently identified in depression, including those involving neurotransmission [60,61].

In addition to their direct neuro-emotional consequences, these biological dysregulations provide an understanding of the extraordinary relationship between depressive symptoms/MDD and subsequent risk of chronic non-communicable diseases such as cardiovascular disease, obesity, diabetes, and neurodegenerative diseases [58]. Psychosocial stress, well known to be associated with depression [62], can provoke low-grade inflammation; however, once initiated, chronic low-grade inflammation appears well-capable of contributing to further depressive symptoms, cognitive impairment, anxiety, fatigue, sleep problems, and pain [63,64]. Neuroprogression is a term used to describe the cumulative shaping of the central nervous system over time; via pathways of biological dysregulation, this process can mediate the persistence of depression and other mental disorders [65].

Socioeconomic disparities are intimately connected to these known biological dysregulations of depression. Since lower socioeconomic status (SES) is accompanied by chronic psychosocial stress and daily hassles, it is perhaps unsurprising that low SES and neighborhood-level deprivation have been associated with significantly higher biomarkers of AL, including inflammation and oxidative stress [66-69]. Neighborhood-level deprivation and social adversity during the prenatal period is now known to be a distinct risk factor for elevated inflammation in adulthood [70].

Maternal exposure to environmental contaminants (discussed in more detail later) may have detrimental effects on oxytocin signaling [71], a neurohypophysial hormone linked to mood and behavior [72]. A Westernized dietary pattern may also compromise maternal care during the early neonatal period, which in turn may have long-lasting consequences [73]. Neighborhood-level disadvantage is linked to lower diet quality [74]. Moreover, serum antioxidant levels increase with SES at the neighborhood level, which might indicate less physiological burden of oxidative stress and/or increased intake of dietary antioxidants typical of diets rich in fruits and vegetables among the more affluent [75,76]. Higher dietary quality in disadvantaged adults is associated with a lower systemic inflammatory burden [77].

### Diet and mental health

Until recently, the relevancy of nutrition to mental health was directed more toward the consequences of gross deficiencies [78]. The notion that more subtle nutrient inadequacies and overall dietary quality could play an important role in mental health was only rarely [79] part of the discourse within mainstream psychiatric literature. Despite clear theoretical mechanisms suggesting that nutrition was an important, yet overlooked, variable [80], psychiatric research remained focused on pharmaceutical interventions and psychological techniques.

However, advances in the emerging field of nutritional psychiatry provide clear evidence that nutrition is an important consideration in short- and long-term neuro-emotional health, particularly in depression [41,81,82]. The brain is reliant upon amino acids, fats, vitamins and minerals, and trace elements to maintain its functional integrity and high energy demands. Due to this, both macro- and micro-nutritional factors have an important influence on neurocognitive function and mental health. Dietary habits influence the functioning of the immune system (thereby limiting or contributing to low-grade inflammation), sustain the antioxidant defence system, and influence neurotrophic factors that otherwise regulate neuronal growth and plasticity [81].

Population studies continue to show that adherence to healthy (sometimes referred to as ‘traditional’) dietary patterns is associated with lowered risk of depressive symptoms, anxiety, and cognitive decline [83-90]. High-quality perinatal nutrition has been positively associated with mental health outcomes and quality of childhood nutrition with academic performance [91-93]. At the intervention level, short-term adoption of traditional dietary patterns has been shown to have a beneficial influence on mood, cognition, and unresolved fatigue [94-96].

In between the epidemiological work and the emerging intervention studies, there are a host of bench studies. Some demonstrate the divergent influence of Westernized dietary patterns (sometimes described as ‘cafeteria’ or ‘fast food’ in animal studies) and traditional diets on behavior and cognition. The former is typically linked to suboptimal cognitive performance and behavioral changes reflective of human anxiety and/or depression [97].

However, because the Westernized dietary pattern is highly palatable, it may attenuate stress and provide a form of ‘self-medication’ [98-100]; indeed, when animals are withdrawn from a cafeteria diet, there are changes in gene expression governing stress physiology [101]. For humans, this would suggest that a transition off the Westernized diet is itself a stressful experience, perhaps made doubly worse when an individual has been reliant upon the diet to mitigate some level of stress.

The interaction between mood and dietary patterns can be complex [102]; however, there is a collection of research showing that humans often increase their consumption of calorie-dense, nutritionally poor ‘comfort foods’ when confronted with psychological stress [103-105]. The potential palliative effect of high fat is demonstrated by the direct infusion of fatty acids in the stomach (that is, bypassing visual, olfactory, and gustatory cues); when researchers do so, they can quickly offset an experimentally induced lowered mood state [106]. Whether for psychological [107] and/or physiological reasons, the draw toward unhealthy foods is often strongly associated with chronic depressive symptoms and psychological distress [108-111].

### Discounting the future

Impulsivity has been strongly associated with depression, and it appears to persist even in remission [112]. In order to bridge the gap between discussions of SES and the environmental context (including nutrition and the built environment) described later, it may be worthwhile to briefly describe the relevancy of delay (or temporal) discounting. Volumes of research show that humans often discount the value of future rewards and instead prioritize smaller immediate rewards. Greater discounting has been associated with impulsivity, depression, obesity, and various unhealthy lifestyle habits [113-117].

The slant toward discounting future rewards is also associated with socioeconomic adversities [118,119]. Stress, cognitive load, lowered mood state, and even physical aspects of the built environment (those outside conscious awareness) may magnify delay discounting [120,121]. For example, respondents are much more likely to discount the value of future financial rewards and opt for smaller immediate gains while answering questions in the vicinity of a fast-food outlet [122]. Individuals residing in neighborhoods with higher concentrations of fast-food outlets are more likely to take smaller immediate rewards over larger future gains [122]. The mere presentation of fast-food imagery may provoke impatience and compromise the mood lift that is typically associated with viewing nature scenes [123]. On the other hand, the very aspects of the urban built environment that may be missing in low SES areas - natural vegetation-rich green areas - may help diminish delay discounting [124,125].

### Natural environments

A number of extensive reviews have examined the potential mental health value of natural environments. These are areas typically defined as those that are *relatively* unchanged or undisturbed by human culture [126], although they can include areas that are designed, manipulated, and sustained by human interventions. In the context of urban settings, this may include gardens, parks, forests, and waterside areas. The reader is directed to detailed reviews covering the ways in which natural environments as whole (and specific elements within them) may buffer stress, improve cognition, facilitate physical activity, encourage social cohesion, and promote overall health and mental well-being [127-129].

A sampling of research shows that greater levels of greenness at the neighborhood level are associated with lower risk of depression and/or anxiety [130-134]. Indeed, one study from New Zealand found that increases in the proportion of useable or total urban green space in proximity to the home was associated with lower rates of anxiety and mood disorder treatment [135]. Mobility research spanning 3 years shows that individuals who move into areas with a higher greenness (*vs*. their previous residence) experience improved mental health [136].

In the current review, attention is drawn to the increasingly robust research concerning natural environments simply as a means to provide a frame of SES reference for the discussions below. Natural environments may be of particular importance to stress reduction and mental health in socioeconomically disadvantaged neighborhoods wherein higher percentages of green space are linked with healthier daytime salivary cortisol patterns and lower perceived stress [137,138]. However, unlike the gradient of depression risk that points toward the disadvantaged, the concentration of urban green space (and/or its perceived safety, accessibility, quality) and local biodiversity is often slanted in the opposite direction, favoring the affluent and less vulnerable [139-145]. Indeed, the absence of natural environments in low SES neighborhoods, especially in urban areas, may mean the *presence* of environmental grey space as described later.

### Microbiota and dysbiosis

Trillions of bacteria (as well as archaea, fungi, protozoa, and viruses) and their genetic material contribute to the human ecosystem. Although it is often stated that the term microbiome is relatively recent, one ‘coined’ coincidental to the burgeoning use of microbial DNA sequencing technologies, this is untrue. Almost 30 years ago, British scientists wrote: ‘*A convenient ecological framework in which to examine biocontrol systems is that of the microbiome. This may be defined as a characteristic microbial community occupying a reasonably well defined habitat which has distinct physio-chemical properties. The term thus not only refers to the microorganisms involved but also encompasses their theatre of activity*.’ [146]. Undoubtedly, microorganisms operating in the human theater of activity, especially the gastrointestinal tract, have been shown to have many distinct physio-chemical properties, including those that may extend to brain structure and function. Emerging evidence suggests that the intestinal microbiota may have a different composition in those with depression *vs*. healthy controls [147,148].

There are multiple pathways by which microbiota may influence brain development, stress physiology, mood, cognition, and behavior. These include, but are not limited to, direct communication with the brain via the vagus nerve, immune-mediated pathways (for example, cytokine production), limitation of oxidative stress, enhancement of nutrient bioavailability and neurotransmitter precursors (for example, tryptophan), and proper maintenance of the gastrointestinal barrier (that is, preventing intestinal permeability and a subsequent cascade of low-grade inflammation) [149]. Although most of the research concerning intestinal microbiota and mental health rests upon rodent studies, a few human studies have provided preliminary evidence that orally administered probiotics or fermented foods may support good mental health [150-156]. The reader is referred to expert reviews for more detail [157,158].

The research concerning microbiota and mental health is highly attractive because it represents a potential link between dietary patterns and environmental considerations. For example, in an experimental study, the transplantation of fecal microbiota from high-fat-diet-fed donor mice into lean mice (raised on standard chow) resulted in altered neurologic function. Interestingly, there were changes in behavior suggestive of anxiety, increased stereotypical behavior, and decreased memory in lean mice upon fecal transfer [159]. Also, the idea that fermented foods may provide enhanced mental health benefit by both direct microbial influence within the intestinal tract and indirect mechanisms through transformation of food chemicals (for example, enhancing nutrient absorption) has been proposed [160].

The lessons learned over the last several decades concerning the hygiene hypothesis - that which suggests the global rise in allergic disease could be related to diminished opportunity for early life exposure to pathogenic microbe exposure via increased hygiene, antibiotics, smaller family sizes, and altered dietary patterns [161,162] - suggest that its overarching theme may also extend to neurocognitive and mental health [163,164]. For example, it was recently reported that higher consumption of fermented foods is associated with lower rates of allergy in children [165], a finding that could be related to mental health as described above [160].

Although intestinal microbes are strongly influenced by dietary factors, they are also a product of environmental bacteria [166-168]. Remarkably, the level of green space and biodiversity of vegetation surrounding one’s residence has been linked with both diversity of select bacteria on the skin, and lower odds of an allergic IgE reaction to common allergens [169,170]. Keeping in mind that environmental biodiversity (for example, variety of plants and birds in a local setting) has been linked to psychological well-being [171-174], it may very well be the case that ‘access’ to microbial diversity may emerge as a matter of environmental justice.

### Grey space and the environmental push

The preponderance of existing research would suggest that the tripod of healthy dietary habits, diversity of microbiota, and access to quality natural environments are in the best interest of positive mental health. The research also suggests that these are overlapping conversations. For example, closer residential proximity to urban green space and greater park access is associated with healthier dietary habits (for example, more fruits, vegetables, whole grains, nuts/beans, and less fast-food, sodium-rich food, and sugar-rich beverages) and lower insulin resistance [175-177]. In higher population density areas, relatively more natural food/specialty stores, fewer convenience stores, and more physical activity resources are associated with higher diet quality [178]. In socioeconomically disadvantaged neighborhoods, lower levels of open space for physical activity have been linked with greater density of fast-food outlets [179]. It is suggested here that for the most vulnerable (socioeconomically deprived individuals and communities), the odds are stacked against a healthy gut microbiome.

An exercise in visualization may help to crystallize the contention. Imagine residing in a socioeconomically deprived community where grey space is the predominant environmental feature. Current research allows for a realistic picture of the dysbiotic environment that might be experienced in this deprived community. At the individual and the neighborhood level, the disparities (and deprivation as a broad term) to be described below may be determined by income, education, social cohesion, racial/ethnic segregation, evaluations of neighborhood esthetic quality, and/or aspects of safety (real and perceived).

It is understood that not all disadvantaged areas are alike; however, the visualized community in this exercise is based largely on how aggregate disadvantage may be encountered in an already developed (Western industrial) nation. As such, it may have some, or even all, of the aforementioned disparities. It is also understood that increases in wealth and socioeconomic status in relation to shifts from traditional lifestyles (along the lines of global trends in diminished contact with biodiversity coincident with urbanization and development) may itself minimize skin and intestinal microbial diversity [180]. However, the discussions here concerning Westernized nations are not without relevance to global regions experiencing a shift from traditional lifestyles and associated increases in wealth [181].

Notwithstanding the heterogeneity of cities, towns, and the neighborhoods in and around them, certain lifestyle habits may be coincident with disadvantage. These may include increased screen time and indoor sedentary behavior [182-187], less sleep [188-190], dietary patterns of high-calorie, low-nutrient-density foods (including ultra-processed, high sodium, additive-rich foods; and/or less fruits and vegetables) [191-196], excess and/or binge alcohol consumption [197,198], and tobacco use [199]. Even if one did not smoke, there would be increased odds of being exposed to second-hand smoke [200].

Whether through changes in dietary patterns and/or less time spent outdoors, the levels of vitamin D might be lower [201-203]. As mentioned earlier, physiological markers such as lower serum carotenoids and red blood cell omega-3 among residents in this neighborhood provides supportive evidence that the absence of high-nutrient foods is translating into diminished phytochemical and essential fat intake [75,76,204]. In addition, specific nutrients such as magnesium intake may be lower [77], thereby removing a layer of nutritional resiliency. Whether through dietary patterns or other lifestyle variables, rates of functional (chronic idiopathic) constipation would be probably be increased, [205] while the accumulation of advanced glycation end products (AGE) in tissue may be higher [206].

The built environment and elements thereof would be increasing the odds that these lifestyle variables and exposures would remain active and reinforced. Specifically, there would likely be disproportionate industrial and commercial activity, heavy transportation routes, bars, liquor stores, convenience stores, fast-food outlets, and tobacco vendors [207-214]. One might have higher odds of being confronted with visual marketing - for example, billboards, sidewalk signage, targeted screen media delivery - that strongly encourages the maintenance of unhealthy lifestyle choices such as fast-food consumption and tobacco use [215-226]. Grey space is therefore, at least in the opinion of this author, not simply less trees and more concrete; it is a completely divergent mental environment.

Perhaps not surprisingly, brand name logo recognition of major fast-food outlets is notably higher among children in lower SES neighborhoods [227]. The density of fast-food outlets within a neighborhood matters. As much as 31% of the variance in excessive fast-food consumption may be attributable to simply living in urban areas with moderate or high density of fast-food outlets [228]. Moreover, living in the vicinity of billboard advertising of snacks and sweet drinks is associated with decreased daily fruit or vegetable consumption [229]. In line with the delay discounting research cited above, evidence indicates that food advertising pushes unhealthy food consumption more effectively when cognitive load is high (that is, distracting mental demands). Moreover, those with low SES backgrounds have a lower susceptibility threshold to advertising’s effects while under cognitive load [230].

The neighborhood-level availability of healthy food and beverage options may also differ [231-233]. A walk through the retail food environment - whether supermarket or convenience store - may provide a different visual experience regarding shelf space devoted to energy-dense, low-nutrient foods. The internal layout of the stores in this neighborhood may be such that less nutritious foods and beverages dominate, perhaps displacing healthy options [234-238]. The variety of products such as sugar-rich beverages may be higher, their prices lower, and their in-store marketing enhanced [239]. The retail environment may provide a seamless match for targeted screen-media-based marketing toward low-nutrient carbohydrates [240].

Low SES is not only associated with psychological distress, it is a predictor of *subsequent* distress among individuals with a longer history of psychological distress. Thus, a vicious cycle can ensue whereby psychological distress can become more persistent over time [241]. Higher levels of daily hassles and major forms of psychological stress, combined with fatigue - a key symptom of depression that has been specifically linked to low SES status [242] - may enhance marketing messages in two key ways:

First, there is experimental and human evidence that unhealthy dietary patterns, those inclusive of so-called comfort foods, may provide a temporary physiological response that could help mitigate psychological stress [98,243-245]. Even though smoking and beyond-moderate alcohol consumption are ultimately detrimental to mood and act as stressors over time [246], they, like highly palatable dietary items, are also used as a means to mitigate stress [247,248]. Second, the appeal of fast-food and ready-to-eat ultra-processed foods (with low-nutrient density) and alcohol could be higher when fatigue, food insecurity, and economic pressures are at the forefront of thought processes [249-253]. In this way, omnipresent marketing messages and the subtle positioning of unhealthy foods within retail settings are like a trap rigged toward the most vulnerable.

The journey through this environment continues. One would also have increased odds of living in a crowded environment [19,254,255], with increased exposure to airborne pollutants [256-258] and other environmental contaminants [259,260]. The burden of heat stress would likely be more pronounced [261]. Exposure to aircraft and traffic noise would ensure that acoustic stress is common [262-264], and this problematic noise might, in turn, increase the odds of depression [265] and a prescription for an anxiolytic [266]. In this neighborhood, a visit to a physician could more easily conclude with a prescription for an antibiotic [267,268]. The likelihood of carrying elevated numbers of *Porphyromonas gingivalis*, an oral bacterium connected to periodontal disease, may be higher [269]. Since the environmental burden of light at night (LAN) pollution is increasingly widespread [270,271], it has become difficult to assess if lower SES urban communities are differentially influenced; however, disruption of normal circadian rhythms might be commonplace [272].

### Dysbiotic drift

Now we can turn our attention to the ways in which this environment might push dysbiosis. Human and/or experimental research shows that acute and cumulative psychological stress (and associated markers of allostatic load) [273-276], environmental pollutants [277-282], crowding [283], acoustic stress [284], heat stress [285], Westernized dietary patterns [286], high fructose and sodium [287], dietary AGE and food additives via processed foods [288,289], lack of colorful dietary phytochemicals [290,291], magnesium deficiency [292], inadequate omega-3 [293], antibiotic administration [294], excess alcohol consumption [295,296], the oral periodontopathogen *P. gingivalis* (when swallowed) [297], tobacco exposure [298,299], sedentary behavior [300], circadian disruptions [301], sleep problems with functional constipation [302], and low levels of vitamin D [303,304] are each individually associated with marked shifts in the intestinal microbiota. The increasing use of artificial sweeteners by socioeconomically disadvantaged and minority communities [305] may also promote dysbiosis [306-308].

It becomes plain to see that at virtually every theoretical turn in which dysbiosis could arise, the socioeconomically disadvantaged may be at higher risk (see the ‘Dysbiotic forces on a socioeconomic gradient?’ section). The collective force of the factors listed above may facilitate a dysbiotic drift among those who have high levels of psychological distress and depressive symptoms. If microbiota are indeed at the intersection of nutrition, environmental health, and lifestyle medicine, as these avenues pertain to mental health, then perhaps it might be time to introduce more detailed discussions of the term disadvantage into the growing gut-brain-microbiota discourse.

### Dysbiotic forces on a socioeconomic gradient?

- Psychological stress
- Processed and ultra-processed foods
- Food additives
- Advanced glycation end products (AGEs)
- Absence of phytochemicals
- Inadequate essential fats
- Inadequate vitamin D
- Inadequate magnesium
- Antibiotic administration
- Potentially pathogenic oral bacteria
- Lack of physical activity/excess indoor screen time
- Tobacco use
- Alcohol use
- Sleep problems/circadian disruptions
- Crowding
- Climatic stress
- Environmental toxins

## Summary and future directions

Despite its obvious ripple into so many aspects of societal health, the ‘No Health without Mental Health’ dictum is often afforded only lip service by funding bodies [309]. The crisis-level mental health challenges on planet Earth are often given a back-seat to fanciful and fantastical research endeavors [310]. Viewed only superficially, mental health disorders cost well over a trillion (USD) per year in the United States and Europe [311,312]. Add to that the interaction between mental illness, substance abuse, and the bloated criminal justice system, and the costs soar even higher [313,314]. Further add the contribution of mental health disorders (and sub-threshold conditions) toward other burdensome NCDs. Consider still the emerging research on trans-generational outcomes of psychological distress [315], and the financial argument for exploring the brain (in its social-environmental-microbial context) becomes painfully obvious.

We are now more than a decade removed from the original (modern era) hypotheses suggesting that the administration of beneficial microbes could have neurocognitive [163] and anti-depressant [316] properties. These original hypotheses suggested a potentially modest, adjuvant role for beneficial microbes. Currently, experts suggest that the gut-brain-microbiota axis represents one of the most exciting areas of research to emerge from the neurosciences [317]. At this point it is unclear how (or if) the current body of research will translate into meaningful evidence that can be used by clinicians and policymakers. However, there now seems to be enough background research to at least anticipate downstream questions that could easily arise.

If microbial therapies did provide benefit in mental health, who would be poised to see the most significant gains? How effective would beneficial microbes be when they are used as an attempt to overcome dysbiotic drift? They may have some benefit, but what would be their staying power? Short-term studies will not answer that question. The administration of select beneficial microbes may indeed provide mental health value, perhaps most especially when provided during the perinatal period. However, their true benefit may be obscured when the environmental context in which they are administered remains unchanged.

Therapeutic microbes for health promotion are often thought of as encapsulated probiotics or material ready for pharmaceutical-grade fecal transplant [318]. Massive financial investments are being made to develop drugs that can combat the dysbiotic pathways associated with chronic NCDs [319]. Hopefully, some of these investments will provide returns. Those discussions, however, mostly avoid the topic of the socio-ecological variables that might set up dysbiosis in the first place. Such variables, if greater attention was paid to them, might preclude the need to administer more laboratory-generated drugs directed at the gut microbiome.

A psychobiotic is currently defined as ‘*a live organism that, when ingested in adequate amounts, produces a health benefit in patients suffering from psychiatric illness*’ [320]. This is a narrowly defined term for several reasons. First, it restricts organisms to their live state. Recent human data, however, shows that heat-inactivated microbes can influence immunity [321] and promote mental health (reduction of tension-anxiety) [322]. Second, it bypasses the notion of prevention and utility in those who might sit under diagnostic criteria (that is, well individuals or those who may not be suffering from an overt psychiatric illness, yet not in optimal mental health as the WHO see it). Third, the definition restricts to ingestion (that is, does not consider inhalation and/or cutaneous routes of influence [323]). Fourth, the term provides no obvious link to the basis of much of the discussion here in this review - internal and external ecosystems. It could be argued that -biotic attached to the prefix eco- would be a better choice; however, that term is now firmly entrenched in commercial trademarked microbial products, and furthermore, it makes no reference to mental health specificity.

The term eco-psychotropic may capture the broad lens from which this topic might be viewed. Eco-psychotropics include any microorganisms, or parts thereof, that can benefit human ecosystems and/or physiology in the promotion of mental health. This allows for both pharmaceutical-oriented discussions and wide-ranging dialogue whereby the *deprivation* of eco-psychotropics considers natural environments and more upstream attempts to address the environmental forces that push toward dysbiosis. Eco-psychotropics could be living and non-living microbes carried within fermented foods; yet they may also be defined as the microbial agents that transform foods during fermentation such that they (the food items) are more capable of promoting mental health [160]. Activities such as community gardening [324], or spending time in natural environments [164], may place one in contact with eco-psychotropics.

Patents, broad intellectual property claims, and commercial interests could easily direct the gut-brain-microbiota research juggernaut toward single microbe/microbial product solutions. The enthusiasm of the psychopharmacologist who might view encapsulated products as a microbial fluoxetine or alprazolam is understandable. Nonetheless, if such directed developments in research are viewed distinctly from glaring environmental factors (socioeconomic, ecological, marketing forces, and otherwise), it might overestimate the potential value of therapeutic outcomes, particularly in the most vulnerable.

There seems little question that environmental factors are upstream and modifiable variables on the course toward sub-threshold and diagnosable mental health disorders. There is no evidence, at least in North America, that the rates of psychological distress are declining [325,326]. Given that medication and psychotherapy interventions provide only modest benefit, and estimated value may be over-inflated by publication bias [327-333], a new frontier of therapeutic hope, one that might act in synergy with first-line interventions, is most welcome. Emerging studies in the clinical setting will undoubtedly help to provide much needed evidence to guide policy and practice [334].

The young science of the microbiome-brain connection has established that microbes matter. If this area of research is to fulfill all of its promise, there must surely be a turn toward the exploration of its relevancy within socioeconomic and other environmental contexts. To date, the words socioeconomic, disadvantaged, deprived, and vulnerable have largely escaped discourse within the emerging studies and primary reviews; medical news stories suggest that the field is turning its attention toward precise clinical evaluations of probiotics in mental health [335]. Now might be an appropriate time to broaden the dialogue with an eye toward who might have those most to gain (or, conversely, those who are likely to gain the least).

Differences in oral salivary microbiota have been noted along socioeconomic lines [336]. It would be interesting to know if there are distinctions in the oral, intestinal, and/or skin microbiota that exist along a neighborhood SES gradient. If so, are those distinctions connected to features of the natural (or built) environment and/or mental well-being?

Many Charters and Constitutions now set forth that humans have a fundamental right to live in an environment that supports their overall health and well-being [337]. Environment is obviously a broad term. It could be argued that an environment filled with visual and auditory cajoling toward unhealthy lifestyle behaviors is at odds with this fundamental right. It could be theorized that diversity of unseen biotic elements - including non-pathogenic microbes - are an essential environmental component in the support of health and well-being; and by extension, the grey space factors that might contribute to dysbiotic drift would also be at odds with this right.
